# Derivation of Respiratory Metrics in Health and Asthma

**DOI:** 10.3390/s20247134

**Published:** 2020-12-12

**Authors:** Joseph Prinable, Peter Jones, David Boland, Alistair McEwan, Cindy Thamrin

**Affiliations:** 1 The School of Biomedical Engineering, University of Sydney, Darlington 2006, Australia; alistair.mcewan@sydney.edu.au; 2 The Woolcock Institute of Medical Research, University of Sydney, Glebe 2037, Australia; cindy.thamrin@woolcock.org.au; 3 The School of Electrical and Information Engineering, University of Sydney, Darlington 2006, Australia; peter.jones@sydney.edu.au (P.J.); david.boland@sydney.edu.au (D.B.)

**Keywords:** asthma, respiratory monitoring, machine learning, U-Net, LSTM

## Abstract

The ability to continuously monitor breathing metrics may have indications for general health as well as respiratory conditions such as asthma. However, few studies have focused on breathing due to a lack of available wearable technologies. To examine the performance of two machine learning algorithms in extracting breathing metrics from a finger-based pulse oximeter, which is amenable to long-term monitoring. Methods: Pulse oximetry data were collected from 11 healthy and 11 with asthma subjects who breathed at a range of controlled respiratory rates. U-shaped network (U-Net) and Long Short-Term Memory (LSTM) algorithms were applied to the data, and results compared against breathing metrics derived from respiratory inductance plethysmography measured simultaneously as a reference. Results: The LSTM vs. U-Net model provided breathing metrics which were strongly correlated with those from the reference signal (all *p* < 0.001, except for inspiratory: expiratory ratio). The following absolute mean bias (95% confidence interval) values were observed (in seconds): inspiration time 0.01(−2.31, 2.34) vs. −0.02(−2.19, 2.16), expiration time −0.19(−2.35, 1.98) vs. −0.24(−2.36, 1.89), and inter-breath intervals −0.19(−2.73, 2.35) vs. −0.25(2.76, 2.26). The inspiratory:expiratory ratios were −0.14(−1.43, 1.16) vs. −0.14(−1.42, 1.13). Respiratory rate (breaths per minute) values were 0.22(−2.51, 2.96) vs. 0.29(−2.54, 3.11). While percentage bias was low, the 95% limits of agreement was high (~35% for respiratory rate). Conclusion: Both machine learning models show strong correlation and good comparability with reference, with low bias though wide variability for deriving breathing metrics in asthma and health cohorts. Future efforts should focus on improvement of performance of these models, e.g., by increasing the size of the training dataset at the lower breathing rates.

## 1. Introduction

There has been increasing interest in monitoring health using wearable sensors. However, very few developers have focused on technologies to monitor the breathing signal. While the ability to monitor breathing daily may be beneficial for tracking general health, it may also be especially relevant for respiratory-related diseases such as asthma and chronic obstructive pulmonary disease (COPD). In asthma, monitoring of lung health is often done using standardised lung function tests such as spirometry and peak expiratory flow; the latter has been shown to be useful for predicting the risk of an asthma episode [1,2]. However, these tests can be difficult to perform for patients, even with the availability of digital spirometers and peak flow meters outside the clinical setting, as it involves forced manoeuvres and training. Although spirometry is important for the diagnosis of COPD, daily monitoring of spirometry/peak flow is considered to be of limited benefit and not commonly used in COPD [3]. The tests are also typically only done once or twice a day, and not suitable for continuous monitoring. In COPD, such tests are of limited benefit and not commonly used [3]. Monitoring respiratory rate has been shown to be useful in predicting upcoming COPD flare-ups [4], but such measurement was only possible via an oxygen monitor in patients who happened to be on home oxygen therapy.

The availability of a non-invasive sensor that measures breathing continuously and in an ambulatory manner would have potentially significant implications on day-to-day disease monitoring. One sensor of interest, and of widespread availability, is the pulse oximeter, which is commonly used in a clinical setting to measure both arterial blood oxygen saturation and heart rate. However, standard commercial pulse oximeters are not suitable for use outside the clinical setting because they require tethered/cabled fixture to the finger and their functionalities are limited during everyday activity, e.g., for walking or exercising, or during daily activities at home. Commercial wearable technologies from companies such as Garmin, Apple, and Fitbit, among others [5], contain pulse oximeters that can continuously monitor respiratory rate, heart rate, and O2 saturation outside a clinical setting, though their accuracy in extracting respiratory rate has not been clinically tested in a large cohort.

One challenge facing reliable application of pulse oximeter sensors is the accurate extraction of the breathing signal from the photoplethysmogram (PPG) signal. State-of-the-art machine learning algorithms have shown early promise in achieving this, in particular the U-shaped network (U-Net) [6] and Long Short-Term Memory (LSTM) architectures [7,8]. In our previous work [8], we demonstrated for the first time that detailed respiratory metrics could also be extracted from a volume trace acquired during normal, tidal breathing using a LSTM network in healthy participants. However, it is unclear how well the U-Net architecture can extract these metrics, nor how either of these methods perform in disease populations.

In this proof-of-concept study, we aimed to determine the feasibility of extracting the breathing signal and respiratory metrics from a PPG signal in both healthy and asthmatic populations, using the U-Net and LSTM machine learning algorithms, and compared the performance of these approaches against a gold standard respiratory band.

## 2. Materials and Methods

### 2.1. Study Subjects

Measurements were recorded from a group of participants (11 healthy and 11 with asthma) who provided informed consent. Subjects were volunteers recruited from the Woolcock Institute of Medical Research database. The protocol for this study was approved by Northern Sydney Local Health District Human Research Ethics Committee (#LNR/16/HAWKE99 ethics approval).

### 2.2. Breathing Data Collection

Participants conducted five randomized breathing serials at a rate of 6, 8, 10, 12, or 14 breaths per minute. Each serial was conducted for five minutes. Each participant was coached to breathe at a specific rate by following a visual prompt shown on a desktop screen. The prompt contained a window that was set to the target time for each breath and a marker showing % through the target time.

An Alice PDx (Phillips Respironics, Murrysville, PA, USA) portable diagnostics system was used to acquire measurements during the day. This provided the reference gold standard volume signal, based on electrical inductance changes arising out of movement detected by two elastic bands of winding coils wrapped around the chest and abdomen, sampled at 100 Hz. The Alice PDx also simultaneously measured the PPG signal sampled at 75 Hz. Both signals were subsequently resampled to 25 Hz to reduce the computational time required to train models.

### 2.3. Principle of Breathing Signal Extraction from the PPG Signal

**Figure 1:**
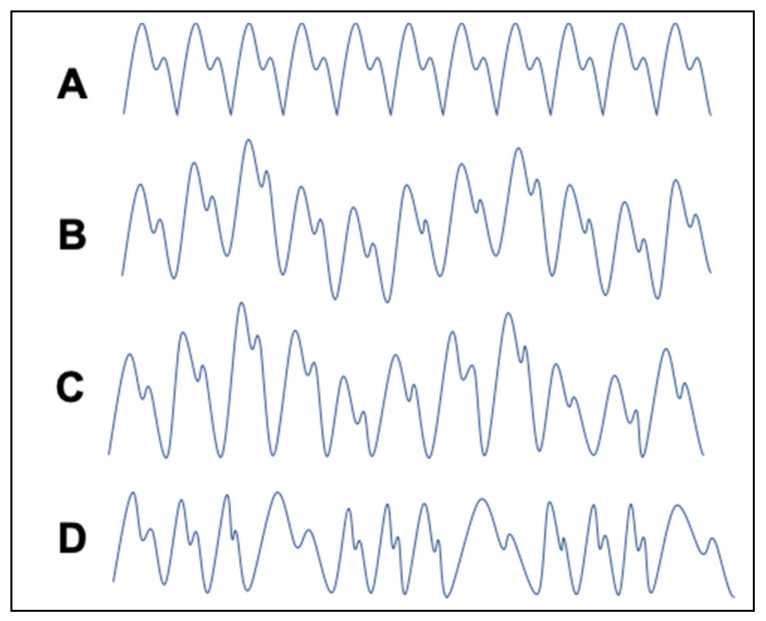
Illustrative examples of the photoplethysmographic signal showing: (**A**) no modulation or wander; (**B**) baseline wander; (**C**) amplitude modulation; (**D**) pulse width modulation potentially induced by breathing cycles.

It is possible to extract the breathing signal from the photoplethysmogram signal (PPG) generated by the pulse oximeter because breathing periodicity [9,10,11] and effort [12] modulate the PPG amplitude, frequency, and baseline wander. This is shown in Figure 1.

### 2.4. Machine Learning Models Used

We compared two machine learning architectures to extract the relative volume trace from the pulse signal: (1) the U-Net architecture, adapted from the original methods described by Rivichandran et al. [6] and (2) an LSTM network, previously described by Prinable et al [8]. The LSTM network is an architecture that has gated connections designed to learn patterns in historical data by regulating information flow, while a U-Net learns patterns by passing information through a series of filters. Both networks were trained on a Dell Optiplex D810, i7, 32 GB RAM, and two Titan Xp (NVIDIA, Santa Clara, CA, USA) graphics cards. Both models were programmed in Python using the Keras open-source library. Each model was trained over multiple epochs with each epoch referring to one run through the whole training dataset. We stopped model training if the performance did not improve after 5 epochs. The dataset was split up using 5-fold cross validation to verify the generalisation of the models.

### 2.5. Extraction of Key Breathing Metrics from Generated Volume Trace

**Figure 2:**
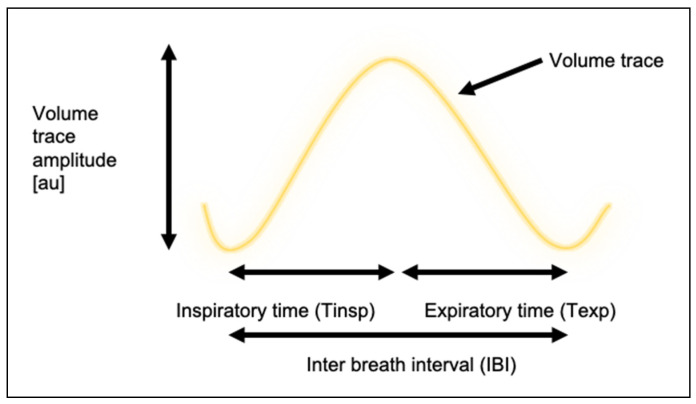
Diagrammatical definitions of respiratory metrics derived from a volume trace.

The output of both the machine learning architectures was a Tidal Volume Waveform (TVW). A Savitzky–Golay filter was used to smooth the trace for both the LSTM and U-Net. We then extracted the following breathing metrics: inspiration period, expiration period, breathing rate, and inter-breath interval. The inspiration to expiration (I:E) ratio was calculated from the inspiration period and expiration period. These metrics are shown in Figure 2.

In this work, we define the previous terms as follows:

- inspiration period (Tinsp): the period in seconds between a trough and a peak within the TVW signal.
- expiration period (Texp): the period in seconds between a peak and a trough within the TVW signal.
- I:E ratio: the ratio between consecutive inspiration time and expiration period. Derived values for Tinsp and Texp are used for this calculation.
- inter-breath interval (IBI): the period in seconds between two consecutive peaks within the TVW signal.
- breathing rate (BR): the amount of breaths per minute (derived independently of IBI).

Since the breathing signal obtained from either the gold standard or the PPG signal is a relative rather than absolute volume trace, i.e., only showing relative changes in volume, we did not consider tidal volume as a metric.

### 2.6. Performance in Extracting Breathing Traces and Metrics

There was a total of 550 windows obtained from 22 participants, 5 breathing rates, and 5 folds of data. In each window, the reference respiratory metric was extracted and compared to both the LSTM and U-Net predictions. In our previous paper [8], we post-processed the output data and excluded windows that did not meet a specific minimum correlation. In this analysis, we used all the available data as a better representation of the accuracy that could be attained if the system was implemented in real time.

### 2.7. Statistical Analysis

The volume trace derived from the pulse oximetry sensor and the gold standard were compared using Pearson correlation coefficients. Extracted breathing metrics were compared against the gold standard using paired t-tests and Bland–Altman analyses.

## 3. Results

### 3.1. Participant Population

The demographic information of the 22 participants is shown in Table 1. Ten (45%) of the participants were male. The mean (standard deviation, SD) for participant age was 43.8 (18.0) years. Eleven participants reported doctor-diagnosed asthma, with optimal asthma control as a group (mean (SD) 5-point Asthma Control Questionnaire (ACQ5 [13]) score 1.04 (0.94)), though 2 of these had sub-optimal asthma control based on ACQ5 > 1.5. Table 1 shows that they have mild airflow limitation and obstruction based on spirometry (%predFEV1 and FEV1/FVC).

### 3.2. Datasets

**Figure 3:**
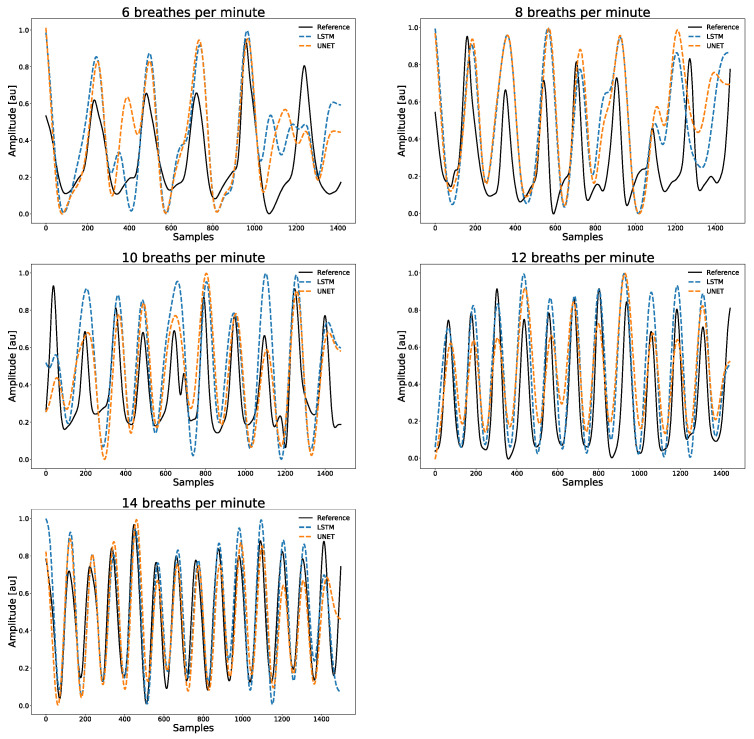
Example window for 6, 8, 10, 12, and 14 breaths per minute is shown for Participant 1.

An example window for 6, 8, 10, 12, and 14 breaths per minute is shown for Participant 1 in Figure 3.

#### Training Time

**Figure 4:**
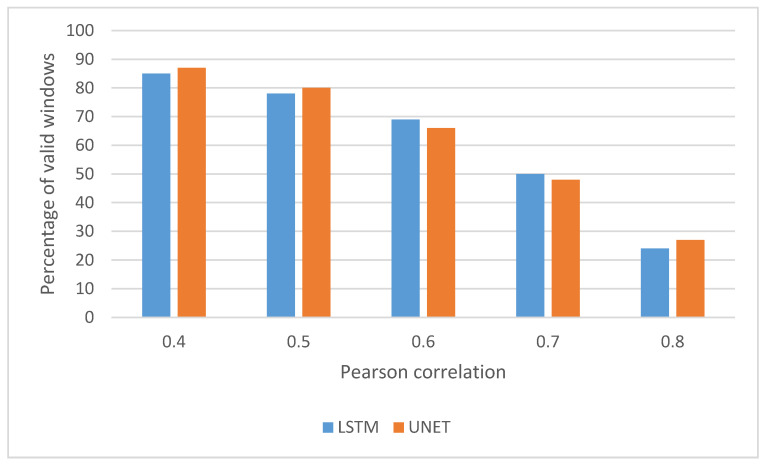
Percentage of valid windows exceeding corresponding Pearson correlation with the reference signal for the Long Short-Term Memory (LSTM) and U-shaped network (U-Net.)

The LSTM trained slower: 18 (3) minutes vs. 11 (4) mins for the U-Net. Both architectures took an input of 320 samples (~13 seconds) and predicted a single sample from the respiratory waveform. Approximately 13 seconds of input data were selected based on previous parameter search optimisation [8]. The results of this comparison led to moderate correlation (r = 0.6) for both networks. The breakdown of windows that exceeded a certain correlation is shown in Figure 4.

The paired t-test between derived and gold standard metrics for all people and respiratory rates are reported in Table 2.

The Pearson correlation and mean bias and 95% limits of agreement (LoA) between derived and gold standard metrics are shown in Table 3.

**Figure 5:**
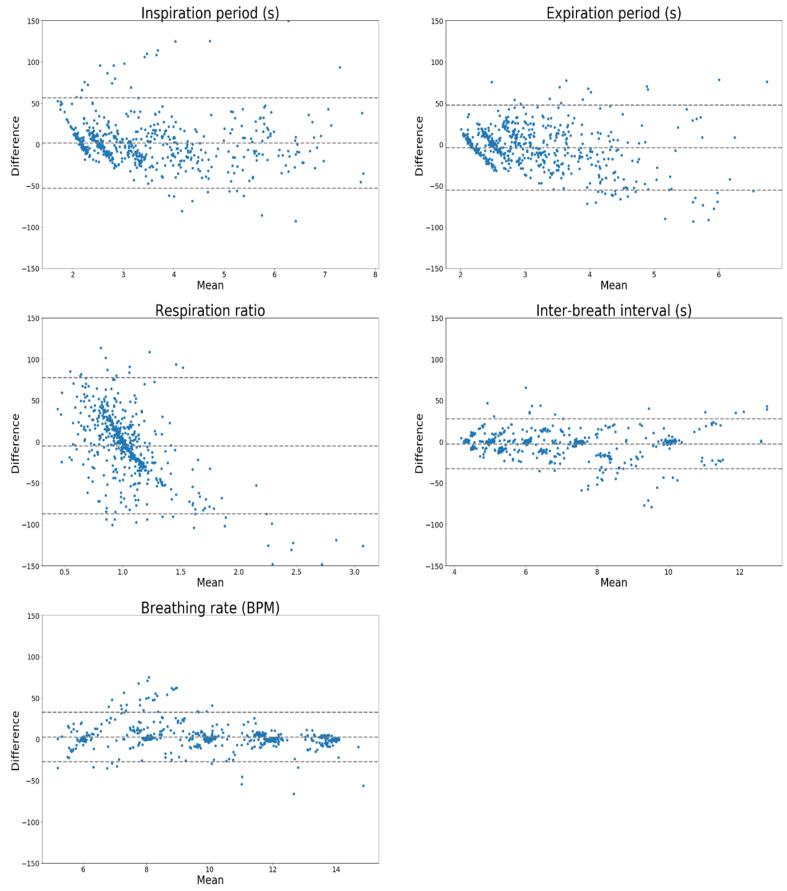
Bland–Altman agreement expressed as percent differences for LSTM architecture.

**Figure 6:**
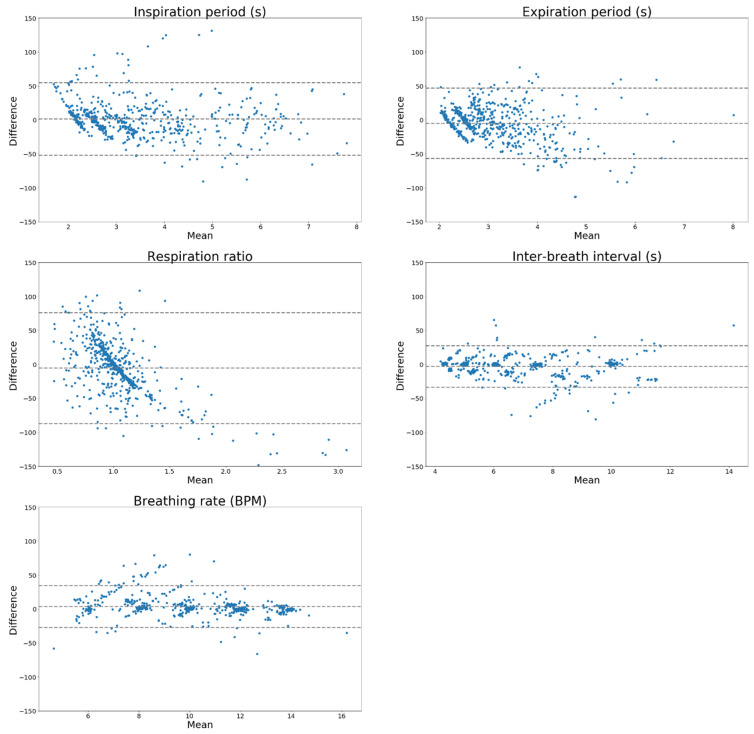
Bland–Altman agreement expressed as percent differences for U-Net architecture.

The Bland–Altman agreement between derived and gold standard metrics for all people and respiratory rates are shown for the U-Net in Figure 5 and LSTM in Figure 6.

## 4. Discussion

This is the first implementation of machine learning methodologies to extract respiratory metrics from a PPG in both asthma and health groups in an ambulatory setting. While the Pearson correlation between the actual and predicted relative tidal waveform was relatively moderate (LSTM r = 0.65, U-Net r = 0.64), the resultant waveforms still contained sufficient information to adequately extract key breathing metrics of inspiration period, expiration period, inter-breath interval, and respiratory rate.

The U-Net showed similar performance to the LSTM in terms of extracted respiratory metrics with the reference signal as shown by the comparable bias for all metrics in Table 2. Both methods provided strong correlations with the gold standard, particularly for breathing rate and inter-breath intervals. However, the variability was very high for most metrics, with limits of agreement up to ±56% (with the exception of I:E ratio which had unacceptable performance overall). Best performance was seen again for the breathing rate and the inter-breath intervals, with limits of agreement up to ~35%.

The Bland–Altman plots (Figure 5 and Figure 6) show evidence of proportional bias, which may be due to the detection of spurious breaths in the extracted volume traces which do not correspond directly to a real breath in the reference volume trace. This may have resulted in large apparent deviations in, e.g., the breathing rate or inter-breath interval compared to the closest available breath from the reference signal; the deviation thus becomes larger with the size of the breathing rate or inter-breath interval itself. The proportional bias appears centred around each of the breathing rates used to train and test both models.

In the current work, the U-Net architecture required more computational power than the LSTM though it took a shorter time to train. If memory requirements are a major consideration in implementing a machine learning approach in a wearable device, then the LSTM should be selected. Otherwise, the U-Net is a better option to reduce training time with a large dataset.

To date, it is unclear whether continuous measures of breathing metrics such as Tinsp, Texp, IBI, and breathing rate are good predictors of an asthma exacerbation. Further, it is unclear as to how often respiratory measures would need to be captured to have a correlation to asthma exacerbation. If performance can be improved, then the limitation to achieving long-term, continuous monitoring of respiratory metrics to evaluate this will no longer be the algorithms themselves but, rather, the implementation of those algorithms on a wearable device. This will provide us with a tool to investigate the clinical utility of ambulatory respiratory monitoring. Furthermore, the data could be made securely accessible to a respiratory patient’s doctor or nurse practitioner in real time for potential early intervention.

An area of concern for long-term monitoring with wearables is compliance. Previously, we found that smart watch technologies are likely to have the highest compliance rate compared to a chest strap or other respiratory monitoring device [14]. It is unlikely that these devices have the processing power to train models though they do have enough computing power to run them.

In practice, regardless of machine learning model used, patient-specific training would be necessary. This could be realised by having the individual wear a chest band in addition to a pulse capture smart watch for an initial “training” period and breathing at a range of respiratory rates, with the chest strap no longer being required after the model was successfully trained. We previously demonstrated that a single model was sufficient to predict respiratory rate for a single participant over the period of a month’s time [6].

Our study has a number of limitations. First, breathing metrics derived with both models showed comparable bias across all breathing rates, but the variability was high particularly for inspiration and expiration times, and the respiratory ratio. The causes for this are unknown, but we noted that variability tended to be higher at the lower breathing rates and may be driven by poor breath detection. This, in turn, may be due to insufficient breaths available for training at these lower breathing rates, since fewer breaths are available during a fixed time period. Subsequent efforts should focus on improving the performance at these lower rates, by increasing the presence of low respiratory rate breathing cycles in the training dataset. Another approach to reduce variability would be to assess the quality of the PPG signal and exclude windows with poor quality [10,15], though this would mean loss of information during, e.g., noisy periods. Nevertheless, the bias and standard deviation presented in this work fall in line with our previous findings [8] and perform better in terms of bias and 95% LoAs for respiratory rate in other studies employing larger datasets [10,16,17,18,19]. The high variability may limit applicability in the clinical setting, but the performance may be adequate for general long-term monitoring of breathing rate for day-to-day use.

Second, the asthma cohort in this study have mild disease based on their lung function (%predFEV1 and FEV1/FVC). Ambulatory breathing patterns in severe asthma have not been well characterised, highlighting the lack of available tools for such investigations. Studies within clinical research laboratory settings have shown, e.g., no differences in variability during acute vs. refractory severe asthma [20], and yet increased variability of inter-breath intervals in some asthma phenotypes [21]. Further work will be needed to determine how the algorithms perform with severity of disease and irregular breathing, as well as patient factors such as age, BMI, and fitness status. However, we propose that may be partly mitigated with appropriate training based on the patient’s own breathing pattern.

In conclusion, this work informs the further development of machine learning models for extracting respiratory metrics from PPG signals, using real-world data from both asthmatic and healthy groups. We have demonstrated that such a modality is feasible, but training the data appropriately may be the key to successful implementation.

## Figures and Tables

**Table 1:** Participant information for the study, stratified by health status.

	Status	
Characteristic	No Asthma (n = 11)	Asthma (n = 11)
Sex: Male n (%)	6 (55)	4 (36)
Age, mean (SD) years	30.1 (7.3)	55.9 (16.3)
BMI, mean (SD) kg/m^2^	25.1 (4.8)	26.7 (5.0)
ACQ5, mean (SD)		1.04 (0.94)
%predFEV1, mean (SD)		84.6 (22.1)
%predFVC, mean (SD)		102.8 (15.9)
FEV1/FVC, mean (SD)%		68.3 (15.3)

ACQ5: 5-point Asthma Control Questionnaire; %predFEV1: Forced expiratory volume, percentage of predicted; %predFVC: Forced vital capacity, percentage of predicted; FEV1/FVC: Forced expired volume/forced vital capacity.

**Table 2:** Paired t-test between derived and gold standard metrics for all people and respiratory rates.

Metric	Reference	LSTM n = 550		U-Net n = 550	
	Mean (SD)	Mean (SD)	*p*-Value	Mean (SD)	*p*-Value
Tinsp (s)	3.50 (1.47)	3.51 (1.38)	*p* = 0.87	3.48 (1.33)	*p* = 0.83
Texp (s)	3.28 (1.19)	3.09 (0.88)	*p* < 0.05	3.04 (0.83)	*p* = 0.001
I:E ratio (unitless)	1.11 (0.62)	0.97 (0.20)	*p* < 0.001	0.96 (0.19)	*p* = 2.63
BR (BPM)	9.99 (2.81)	10.21 (2.53)	*p* = 0.17	10.28 (2.52)	*p* = 0.07
IBI (s)	6.77 (2.15)	6.59 (2.05)	*p* = 0.14	6.52 (1.99)	*p* < 0.05

**Table 3:** Derived breathing metrics using the LSTM and U-Net methods and associated statistical analyses.

Method	r^2^	*p*	Absolute Bias	95% LoA	Relative Bias (%)	95% LoA
Tinsp (seconds)						
LSTM	0.66	*p* < 0.001	0.01	−2.31 to 2.34	1.89	−52.95 to 56.74
U-Net	0.69	*p* < 0.001	−0.02	−2.19 to 2.16	1.30	−52.15 to 54.74
Texp (seconds)						
LSTM	0.46	*p* < 0.001	−0.19	−2.35 to 1.98	−3.70	−55.21 to 47.80
U-Net	0.47	*p* < 0.001	−0.24	−2.36 to 1.89	−4.97	−56.84 to 46.89
I:E ratio						
LSTM	−0.04	0.39	−0.14	−1.43 to 1.16	−4.65	−87.18 to 77.88
U-Net	0.01	0.89	−0.14	−1.42 to 1.13	−5.30	−87.07 to 76.47
IBI (seconds)						
LSTM	0.81	*p* < 0.001	−0.19	−2.73 to 2.35	−2.39	−32.76 to 27.97
U-Net	0.81	*p* < 0.001	−0.25	−2.76 to 2.26	−3.16	−33.69 to 27.36
BR (BPM)						
LSTM	0.87	*p* < 0.001	0.22	−2.51 to 2.96	2.99	−27.04 to 33.02
U-Net	0.86	*p* < 0.001	0.29	−2.54 to 3.11	3.69	−27.17 to 34.56
